# Twelve years of circulatory extracorporeal life support at the University Medical Centre Utrecht

**DOI:** 10.1007/s12471-021-01552-z

**Published:** 2021-03-06

**Authors:** C. L. Meuwese, J. A. Hermens, M. de Haan, S. A. Braithwaite, F. Ramjankhan, M. P. Buijsrogge, N. de Jonge, J. H. Kirkels, M. de Jong, W. Pasma, J. L. P. Vromen-Wijsman, A. O. Kraaijeveld, E. E. de Waal, E. Torn, M. Platenkamp, J. J. van der Heijden, O. L. Cremer, D. van Dijk, D. W. Donker

**Affiliations:** 1 Department of Intensive Care Medicine, University Medical Centre Utrecht, Utrecht, The Netherlands; 2 Department of Anaesthesiology, University Medical Centre Utrecht, Utrecht, The Netherlands; 3 Department of Cardiothoracic Anaesthesiology, University Medical Centre Utrecht, Utrecht, The Netherlands; 4 Department of Cardiothoracic Surgery, University Medical Centre Utrecht, Utrecht, The Netherlands; 5 Department of Cardiology, University Medical Centre Utrecht, Utrecht, The Netherlands

**Keywords:** Cardiogenic shock, Extracorporeal life support, ECLS, Mortality

## Abstract

**Introduction:**

Circulatory extracorporeal life support (ECLS) has been performed at the University Medical Centre Utrecht for 12 years. During this time, case mix, indications, ECLS set-ups and outcomes seem to have substantially changed. We set out to describe these characteristics and their evolution over time.

**Methods:**

All patients receiving circulatory ECLS between 2007 and 2018 were retrospectively identified and divided into six groups according to a 2-year period of time corresponding to the date of ECLS initiation. General characteristics plus data pertaining to comorbidities, indications and technical details of ECLS commencement as well as in-hospital, 30-day, 1‑year and overall mortality were collected. Temporal trends in these characteristics were examined.

**Results:**

A total of 347 circulatory ECLS runs were performed in 289 patients. The number of patients and ECLS runs increased from 8 till a maximum of 40 runs a year. The distribution of circulatory ECLS indications shifted from predominantly postcardiotomy to a wider set of indications. The proportion of peripheral insertions with or without application of left ventricular unloading techniques substantially increased, while in-hospital, 30-day, 1‑year and overall mortality decreased over time.

**Conclusion:**

Circulatory ECLS was increasingly applied at the University Medical Centre Utrecht. Over time, indications as well as treatment goals broadened, and cannulation techniques shifted from central to mainly peripheral approaches. Meanwhile, weaning success increased and mortality rates diminished.

**Supplementary Information:**

The online version of this article (10.1007/s12471-021-01552-z) contains supplementary material, which is available to authorized users.

## What’s new?

- Extracorporeal life support (ECLS) for circulatory indications has been used at the University Medical Centre Utrecht for 12 years, during which 347 runs were performed in 289 patients.
- Throughout the 12 years, ECLS indications became more diverse and system set-ups changed, with an increase in peripheral cannulation techniques and in the application of left ventricular unloading techniques as well as selective distal perfusion cannulas.
- Mortality gradually decreased over time, which was likely due to improved patient selection.

## Introduction

Refractory cardiogenic shock has traditionally been associated with high mortality rates [1, 2]. With the evolvement of venoarterial extracorporeal life support (ECLS) in the latter part of the previous century, the arsenal of treatment options was significantly extended. Early disappointing results [3], however, rendered physicians reluctant to apply ECLS in clinical practice.

With the development of more advanced devices incorporating novel centrifugal pumps and biocompatible membranes [4], interest in circulatory ECLS was renewed. Initially, circulatory ECLS was primarily used in paediatric patients with postcardiotomy cardiogenic shock [5]. Subsequently, its use grew to encompass similar indications in the adult population [6]. The preference for ECLS [7] was catalysed by findings from the IABP-SHOCK-II trial demonstrating an absence of mortality benefit of the intra-aortic balloon pump in the setting of cardiogenic shock [8].

In the Netherlands, a number of centres apply ECLS for circulatory and respiratory indications. The University Medical Centre Utrecht (UMCU) has been using ECLS since 2007 in several set-ups, traditionally in the context of cardiogenic shock. Over 12 years, we have seen considerable changes in patient population, indications, complications and outcomes. Except for individual case reports [9], these data have not yet been summarised.

In order to reflect on and to learn from these first 12 years of ECLS in our centre, we performed a retrospective cohort study to describe these characteristics and their evolution over time.

## Methods

A cohort study was performed by retrospectively collecting data from all patients who were supported with venoarterial ECLS for circulatory indications between April 2007, when the first patient was supported, and December 2018 at the UMCU. A waiver was obtained from the medical ethics committee for the retrieval of anonymised data. Information about demographics, comorbidities, ECLS indications, set-up and mortality was collected. Registration of comorbidities occurred on the basis of written diagnoses in the medical chart and included previous presence of diabetes mellitus, chronic kidney disease, peripheral artery disease, transient ischaemic attack (TIA) or ischaemic cerebrovascular accident (ICVA), acute (on chronic) heart failure, coronary artery disease, or previous revascularisation via coronary artery bypass grafting or percutaneous coronary intervention.

Survival status and death causes were recorded at 30 days, hospital discharge, and 1 year after ECLS initiation and at the last time of contact. Patients were censored at each time point when they were alive.

### Indications

ECLS was initiated in patients with circulatory shock, which was defined as systolic blood pressure < 90 mm Hg and/or evidence of insufficient organ perfusion refractory to inotropic and vasopressor support. Circulatory indications included postcardiotomy cardiogenic shock (within 24 h after cardiac surgery), shock due to myocardial infarction, refractory ventricular fibrillation/tachycardia, acute on chronic heart failure, myocarditis or other causes such as pulmonary embolism, septic cardiomyopathy or left ventricular assist device (LVAD) failure. Finally, ECLS was used in patients after lung transplantation as elective extended measure to prevent pulmonary congestion in those with pre-existing pulmonary hypertension or in case of severe right ventricular failure.

The onset and conclusion of an ECLS run were defined by cannulation and decannulation, respectively. A new run was marked by the start of recannulation when a patient had been decannulated or when cannula positions were changed because of a change in ECLS indication (for example, a patient would have received venovenous ECLS for acute respiratory distress syndrome, but cannulas were changed to a venoarterial or venoarterial-venous configuration because of newly developed septic or cardiogenic shock) or due to complications resulting from the previous set-up (e.g. change of cannula position from femoral vein to jugular vein because of thrombosis). This definition was used to maximise the detection of complications and to learn from different ECLS strategies that were used in our clinical practice.

### Set-up extracorporeal life support

The ECLS systems used at the UMCU encompassed a variety of circuit set-ups, all including an extracorporeal centrifugal pump with or without an oxygenator, heater and cannulas. In our clinical practice, Permanent Life Support (Getinge Maquet, Rastatt, Germany), CentriMag (Levitronix-Thoratec-Abbott, Abbott Park, IL, USA) and Cardiohelp (Getinge Maquet) were used. Central cannulation was performed by a cardiothoracic surgeon in the operating theatre, whereas peripheral cannulation was primarily utilised by an interventional cardiologist and/or intensivist in the catheterisation laboratory.

Cannulation of the central aorta was performed using a 20–24–French (F) Elongated One-Piece Arterial Cannula (Medtronic, Minneapolis, MN, USA) or a 20‑F Jostra cannula in combination with an 8‑mm Dacron prosthesis. For right atrial cannulation, a 34- or 36‑F cannula was used; for peripheral cannulation purposes, a 21–25–F Maquet HLS multistage drainage cannula and a 15–19–F Bio-Medicus (Medtronic) arterial single stage cannula were used. An 8‑F Arrow sheath was inserted for selective distal perfusion in case of angiographic obliteration of peripheral arterial flow or signs of limb ischaemia. From 2018 on forward, a distal cannula was also put in place for prevention of ischaemia.

As an adjunct to ECLS, LV unloading strategies were applied when complications of high afterload were seen or anticipated upon ECLS initiation. These included: (a) presence of pulmonary oedema, (b) LV distention and/or virtual absence of LV ejection, (c) pulmonary capillary wedge pressures > 15 mm Hg, or (d) the anticipation of a high risk for development of pulmonary oedema during the ECLS run. An intra-aortic balloon pump was the main technique of choice. When theoretical considerations prior to placement led us to believe that venting capacity of the intra-aortic balloon pump was probably insufficient [10], the left atrium or ventricle was directly vented.

### Statistical analyses

The population was divided into six groups based on the year of presentation, with each group comprising a 2-year time frame. Sequential Organ Failure Assessment (SOFA) scores were calculated by summarising the scores from six subdomains (central nervous system, circulatory, respiratory, coagulation, renal and liver). The central nervous system domain score was missing in some cases due to usage of sedatives just before ECLS initiation. These missing values were imputed with the nearest value, a common technique used for imputation of missing SOFA scores at baseline [11]. Survival After Veno-arterial Extracorporeal membrane oxygenation (SAVE) scores were calculated based on the validated formula published by Schmidt et al. [12]. Acute Physiology and Chronic Health Evaluation (APACHE) IV scores were similarly calculated [13].

Baseline and ECLS characteristics are presented as mean with standard deviation (SD) or as median with interquartile range (IQR), depending on the variable distribution. Baseline characteristics and survival were compared on a patient level and ECLS characteristics on the level of the ECLS run. Trends and differences across groups were compared using linear regression analyses and chi-squared tests for continuous and categorised data, respectively.

To analyse the association between year of first ECLS exposure and mortality, Cox proportional hazard models were constructed. A crude model was composed of year of ECLS initiation and 30-day, 1‑year or overall mortality. In a second model, age, sex and comorbidities were added as covariates. In a third and fourth model, SOFA scores and APACHE IV scores were added, respectively. In separate models, SAVE scores were added as covariate.

In order to compare the observed number of deaths during hospital stay with expected mortality figures as based on the SAVE sore, we computed standardised mortality ratios (SMRs). SMRs are calculated by dividing the observed in-hospital death rate by the hypothetical mortality rate as predicted by the SAVE score. The result is a ratio with 95% confidence interval for each year category.

*P*-values < 0.05 and confidence intervals not including 1 were considered statistically significant. Analyses were performed with RStudio: Integrated Development for R (RStudio, Inc., Boston, MA, VS).

## Results

### Demographics

In total, 347 ECLS runs were performed in 289 patients. Numbers of patients and ECLS runs increased over the inclusion period (Tab. 1). Patients had a mean age of 52 years (SD 16) and were predominantly men (59.5%). A minority of patients had a previous medical history of diabetes mellitus (12%), hypertension (22%), TIA/ICVA (6%) or chronic kidney disease (5%). Mean age slightly increased over time, although this change was not statistically significant.

**Table 1:** Baseline characteristics across 12 years

Variable	Total	2007–2008	2009–2010	2011–2012	2013–2014	2015–2016	2017–2018	*P*-value
Patients	289	14	30	50	71	67	57	NA
Runs	347	16	36	59	79	86	71	NA
Age, years	52.2 ± 15.5	47.4 ± 19.6	49.8 ± 12.3	51.0 ± 15.6	57.3 ± 13.7	57.3 ± 16.4	54.9 ± 16.6	0.450
Men	172 (59.5)	9 (64.3)	18 (60)	30 (60.0)	40 (56.3)	45 (67.2)	30 (52.6)	0.671
BMI, kg/m^2^	25.0 ± 4.8	23.2 ± 3.0	23.7 ± 6.1	25.2 ± 3.7	24.7 ± 4.2	25.0 ± 5.1	26.3 ± 5.7	0.658
DM	35 (12.1)	2 (14.3)	2 (6.7)	6 (12.0)	8 (11.3)	10 (14.9)	7 (12.3)	0.920
Hypertension	63 (21.8)	2 (14.3)	6 (20.0)	10 (20.0)	21 (29.6)	11 (16.4)	13 (22.8)	0.552
CKD	14 (4.8)	1 (7.1)	2 (6.7)	3 (6.0)	4 (5.6)	2 (3.0)	2 (3.5)	0.884
TIA/ICVA	18 (6.2)	0	3 (10.0)	2 (4.0)	5 (7.0)	2 (3.0)	6 (10.5)	0.414

Data are *n*, mean ± standard deviation, or *n* (%)

*SD* standard deviation, *BMI* body mass index, *DM* diabetes mellitus, *CKD* chronic kidney disease, *TIA* transient ischaemic attack, *ICVA* ischaemic cerebrovascular accident, *NA* not applicable

### Technical characteristics

**Fig. 1:**
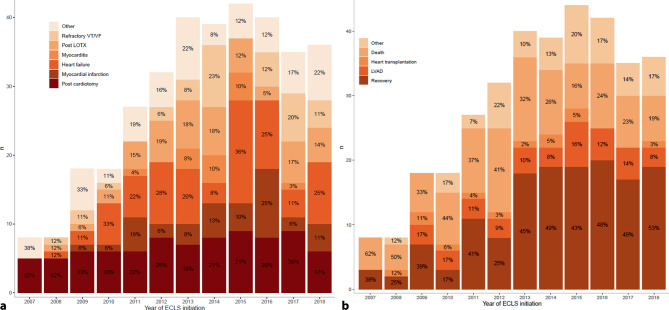
Indications for **a** initiation and **b** cessation of extracorporeal life support (*ECLS*). *VT/VF* ventricular tachycardia/fibrillation, *LOTX* lung transplantation, *LVAD* left ventricular assist device

Postcardiotomy cardiogenic shock (24%) and acute (on chronic) heart failure (21%) were the overall most common ECLS indications, followed by post-lung transplant indications and circulatory shock due to myocardial infarction, refractory ventricular fibrillation/tachycardia and myocarditis. The distribution of indications changed over time (Fig. 1a).

Additionally, central cannulation techniques were caught up by a much larger proportion of peripheral insertions (Tab. 2). Application of LV unloading techniques at initiation increased from nearly 2% to 23% in 2017–2018. An intra-aortic balloon pump (*n* = 33) was primarily used. In 6 other occasions, venting of the left atrium or ventricle was performed and 3 patients were treated with alternative techniques. Moreover, a distal perfusion cannula was increasingly used over the years, but was in general applied in a minority of patients. SOFA scores gradually but significantly decreased over time, while APACHE IV and SAVE scores did not significantly change over time (Tab. 2).

**Table 2:** Characteristics of ECLS runs across 12 years

Variable	Total	2007–2008	2009–2010	2011–2012	2013–2014	2015–2016	2017–2018	*P*-value for trend^a^
Runs, *n*	347	16	36	59	79	86	71	
*Disease severity*								
– SOFA score	10 ± 3.5	NA	11 ± 3.8	11 ± 3.2	10 ± 3.8	10 ± 2.9	8 ± 3.5	<0.001^b^
– APACHE IV score	68 (52–92)	NA	74 (66–102)	67 (51–98)	65 (53–90)	69 (51–85)	66 (52–83)	0.114^b^
– SAVE score	−1.7 (−5.7 to 1.3)	NA	−0.7 (−6.7 to 0.3)	−2.7 (−6.2 to 1.3)	−0.7 (−4.7 to 2.3)	−1.7 (−4.7 to 1.8)	−2.2 (−5.5 to 1.3)	0.214
*ECLS settings*								
– ECLS modus								<0.001
a. VA	312 (91.2)	13 (81.2)	30 (83.3)	56 (94.9)	75 (94.9)	75 (92.6)	63 (88.7)	
b. RVAD	30 (8.8)	3 (18.8)	6 (16.7)	3 (5.1)	4 (5.1)	6 (7.4)	8 (11.3)	
– LV unloading^b^	42 (12.1)	6 (3.8)	6 (1.7)	5 (8.5)	2 (2.5)	7 (8.1)	16 (22.5)	<0.001
– Distal cannula^b^	34 (9.8)	0	1 (2.8)	3 (5.1)	12 (15.2)	6 (7.0)	12 (16.9)	<0.001
– Surgically placed	280 (80.1)	15 (93.8)	34 (94.4)	55 (93.2)	69 (87.3)	65 (75.6)	42 (59.2)	<0.001
*ECLS times*								
– ECLS duration, days	4.2 (1.5–9.6)	3.8 (1.7–7.0)	4.0 (1.5–10.3)	5.1 (1.5–10.3)	3.9 (1.4–8.3)	3.9 (1.4–8.3)	4.1 (1.6–9.2)	0.159
– ICU admission duration, days	11.1 (5.2–26.0)	6.3 (2.8–10.9)	13.2 (2.2–23.2)	11.7 (5.3–24.5)	9.0 (5.5–21.7)	12.1 (6.7–28.3)	12.7 (6.4–27.5)	0.461

*ECLS* extracorporeal life support, *SOFA* Sequential Organ Failure Assessment, *APACHE* Acute Physiology and Chronic Health Evaluation, *SAVE* Survival After Veno-arterial Extracorporeal membrane oxygenation, *VA* venoarterial, *RVAD* right ventricular assist device, *LV* left ventricular, *ICU* intensive care unit, *NA* not applicable

Data are *n*, mean ± standard deviation, median (IQR), or *n* (%)

^a^ *P*-value for trend was calculated using linear or logarithmic regression analyses for continuous and categorised binary data, respectively

^b^ Linear regression analysis with logarithmically transformed APACHE IV scores

In 146 instances (42.1%), ECLS could be successfully weaned. Another 39 (11.2%) and 12 ECLS runs (3.5%) finally ended in LVAD implantation or heart transplantation, respectively. Death during ECLS occurred in 101 patients (29.1%). Because of changes in cannula position and ECLS complications, 37 (10.7%) and 12 (3.5%) runs were ended, respectively. Over 12 years, weaning success increased relative to a decrease in the number of patients dying during support (Fig. 1b).

Median duration of ECLS was 4.2 days (IQR 1.5–9.6) and intensive care unit (ICU) admission lasted a median of 11.1 days (5.2–26.0) (Tab. 2). Both ECLS duration and ICU admission duration did not significantly change over time.

### Mortality

During the total follow-up duration, 181 out of 289 patients (62.6%) died. Of these 181 deaths, 101 (55.8% of the total number of deaths and 34.9% of the total population) occurred during ECLS. After ECLS cessation but before hospital discharge, another 38 patients (21.0%) died. Between hospital discharge and 1 year of follow-up, 20 subjects (11.0%) died. After 1 year, 22 more patients (12.2%) died.

**Fig. 2:**
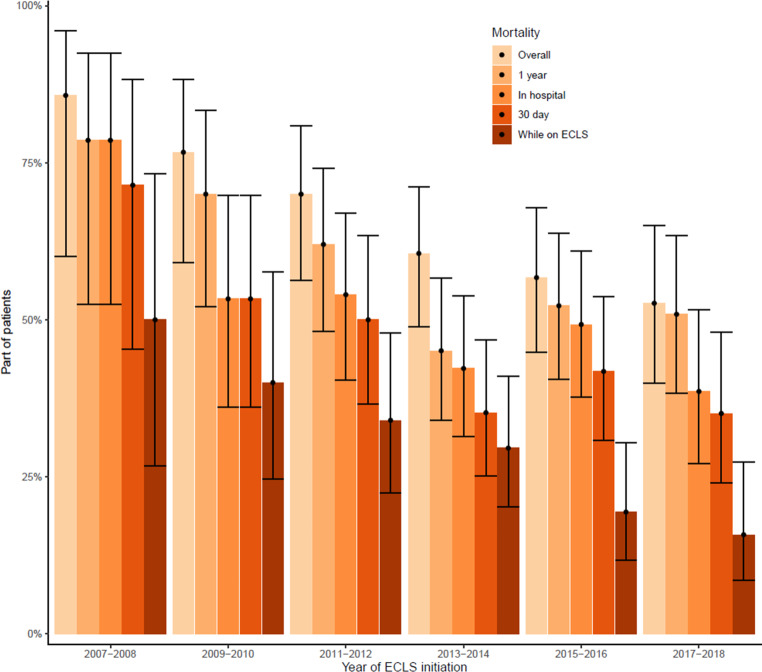
Mean mortality rates (and 95% confidence interval) across years of inclusion. 95% confidence intervals are based on standard error of the mean, as calculated by Wilson’s method.* ECLS* extracorporeal life support

Overall mortality significantly decreased in the period 2007–2008 (86%) compared with 2017–2018 (53%, *p* = 0.002) (Fig. 2). In addition, significant decreases in mortality were noted during ECLS (*p* < 0.001), within the first 30 days (*p* = 0.009) and within 1 year (*p* = 0.017) after ECLS initiation. When analysing the association between year of inclusion and mortality with Cox proportional hazard models, the strength of associations diminished after adjustment for age, sex and comorbidities (Model 2), SOFA score (Model 3) and APACHE IV score (Model 4) (Tab. 3). In those who died while on ECLS, median time to death did not significantly change over the inclusion period (*p* = 0.647) (see Fig. 5 in Electronic Supplementary Material).

**Table 3:** Crude and multivariable adjusted Cox proportional hazard models showing the association between year of inclusion and mortality

Mortality	Crude (Model 1)	Model 2^a^	Model 3^b^	Model 4^c^
30 days	0.93 (0.86–1.00)	0.92 (0.85–1.00)	0.96 (0.88–1.04)	1.01 (0.90–1.09)
1 year	0.94 (0.88–1.01)	0.94 (0.87–1.00)	0.97 (0.90–1.04)	0.98 (0.90–1.06)
Overall	0.96 (0.89–1.02)	0.95 (0.88–1.01)	0.97 (0.91–1.05)	0.98 (0.91–1.06)

^a^ Model 1 plus age, sex and comorbidities

^b^ Model 2 plus Sequential Organ Failure Assessment (SOFA) score

^c^ Model 3 plus Acute Physiology and Chronic Health Evaluation (APACHE) IV score

**Fig. 3:**
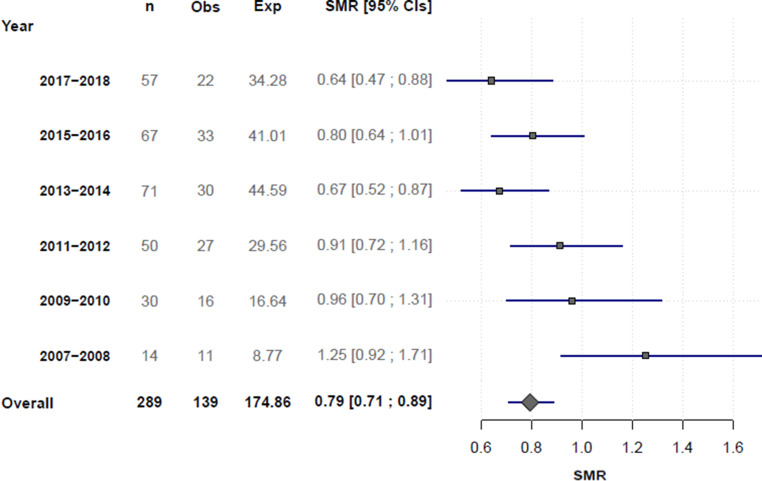
Forest plot showing standardised mortality ratios (*SMRs*) of observed (*Obs*) versus expected (*Exp*) mortality. *CI* confidence interval

Fig. 3 illustrates SMRs of observed versus expected in-hospital mortality rates as predicted by the SAVE score for each category. From 2013–2014 on forward, a dose-response association was seen with gradually lower mortality rates than predicted.

### Causes of death

**Fig. 4:**
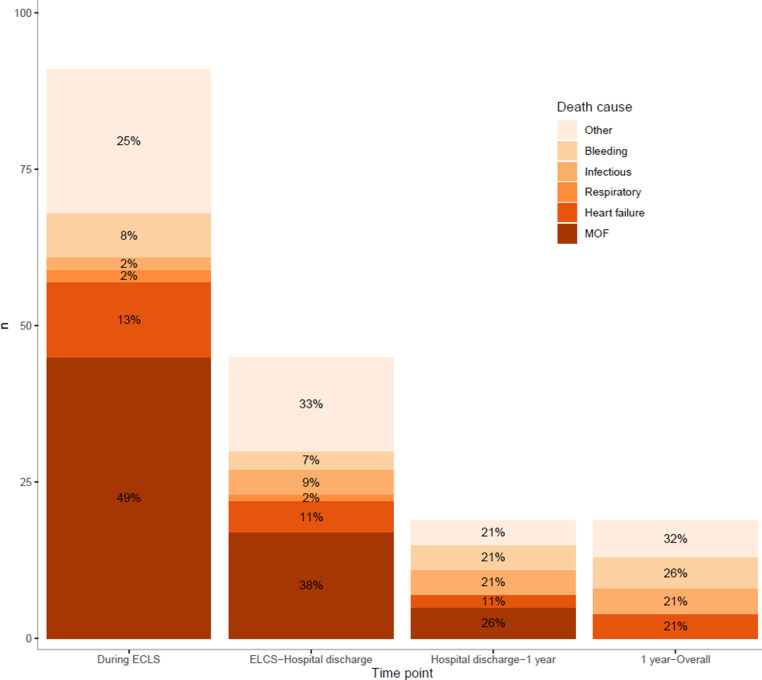
Death cause across phase of recovery. *ECLS* extracorporeal life support, *MOF* multiorgan failure

Death causes were known in 174 deaths. Of these, 67 (38.5%) occurred due to multiorgan failure. Another 23 (13.2%), 14 (8.0%) and 19 (10.9%) patients died as a consequence of refractory heart failure, infectious complications or bleeding, respectively; 51 (29.3%) patients died of other causes. The distribution of death causes significantly changed throughout the different phases of follow-up (*p* = 0.004) (Fig. 4).

The most frequent death cause was multiorgan failure between cessation of ECLS and hospital discharge (see Fig. 6 in Electronic Supplementary Material). Thereafter, bleeding caused the greatest proportion of death. Death causes did not significantly change over time.

## Discussion

Our study, which is based on one of the largest ECLS cohorts in the Netherlands [14–17], describes changes in case mix, indications, set-ups and outcomes over a 12-year period. Within this time frame, ECLS indications broadened and circuit set-ups shifted from central to mainly peripheral approaches. Meanwhile, the rate of successful weaning from ECLS increased and mortality rates diminished. This latter observation could potentially be explained by an altered, and possibly, improved patient selection.

Our data reflect the evolution of ECLS in our centre over time. At first, ECLS was primarily used for refractory postcardiotomy cardiogenic shock. During this phase, patients were centrally cannulated and mortality was high. After this initial phase, ECLS was increasingly applied as bridge to LVAD implantation or heart transplantation. After recognising that a considerable number of patients could be weaned from temporary support, the technique was increasingly deployed as bridge to recovery. With our overall weaning success rate (42%) being comparable to that of other centres, success rates increased up to 53% in the last year of inclusion. During this last phase, ECLS set-ups were refined with a rising use of distal cannulas and LV unloading devices.

The growth in application of LV unloading devices over time (2% to 23%) likely represents an increased recognition of: (a) the relatively high incidence of LV distention [18], and (b) its potential negative impact on the recovery of the left ventricle and on patient recovery [19, 20]. Despite an increase in application of LV unloading at our centre, the highest percentages in 2017–2018 still seem lower than that of other centres [18]. This discrepancy could be explained by different thresholds for placement of an LV unloading device. In certain centres, insertion of ECLS is routinely accompanied by placement of an intra-aortic balloon pump [21], while others only use the combined set-up in selected patients who developed pulmonary oedema [22]. Optimal indications and timing for LV unloading remain for now unknown.

Similar to observations in other ECLS-treated populations [12], and specifically in those after acute myocardial infarction [23] or cardiac arrest [24], we noted a statistically significant decrease in mortality rates over time. Because adjustment for SOFA, APACHE IV and SAVE scores largely abolished the observed association between year of inclusion and mortality, this improvement in mortality rates may reflect better patient selection rather than just evolvement of ECLS management. Nonsignificant changes attributable to better ECLS care may, however, not be excluded, especially since more patients could be weaned from support.

Comparisons between observed and expected mortality rates, as predicted by the SAVE score, suggested improved survival after 2012 as compared with international references. Although it is tempting to attribute this discrepancy solely to good clinical practice at our centre, it should be interpreted with caution for several reasons. First, the SAVE score was designed with data from 2003–2013. In the years thereafter, knowledge likely increased and possibly led to better management. Second, as ECLS has traditionally been applied in the context of LVAD and heart transplant care at the UMCU, patient selection could have biased direct comparisons.

### Limitations

Although our analyses were based on one of the largest ECLS populations in the Netherlands, providing a unique historical overview of ECLS evolution, some limitations apply. First, the association between time of inclusion and overall mortality could have been subject to bias. Follow-up was naturally longer when patients were included earlier and, thereby, the chance of dying could be higher. Nevertheless, in-hospital, 30-day and 1‑year mortality significantly improved over time as well. Furthermore, it is important to consider that our study was performed in a retrospective way. Nevertheless, the primary goal of our study was to describe evolutions throughout history, for which our cohort provided excellent data.

## Conclusion

ECLS is increasingly utilised at the UMCU for circulatory support. Over the course of 12 years, ECLS indications broadened and circuit set-ups shifted from centrally to peripherally cannulated approaches. Meanwhile, the weaning success rate from ECLS increased and mortality rates diminished. This latter observation could potentially be explained by altered, and possibly, improved patient selection.

## Supplementary Information

Supplement, figure 5. Time to death (medians with interquartile ranges) and year of ECLS commencement

Supplement, figure 6. Causes of death across year of ECLS initiation
