# Supplementary figures and images for: Twelve years of circulatory extracorporeal life support at the University Medical Centre Utrecht
Source: Neth Heart J. 2021 Mar 6;29(7-8):394–401. doi: 10.1007/s12471-021-01552-z (PMC8271054; doi:10.1007/s12471-021-01552-z)

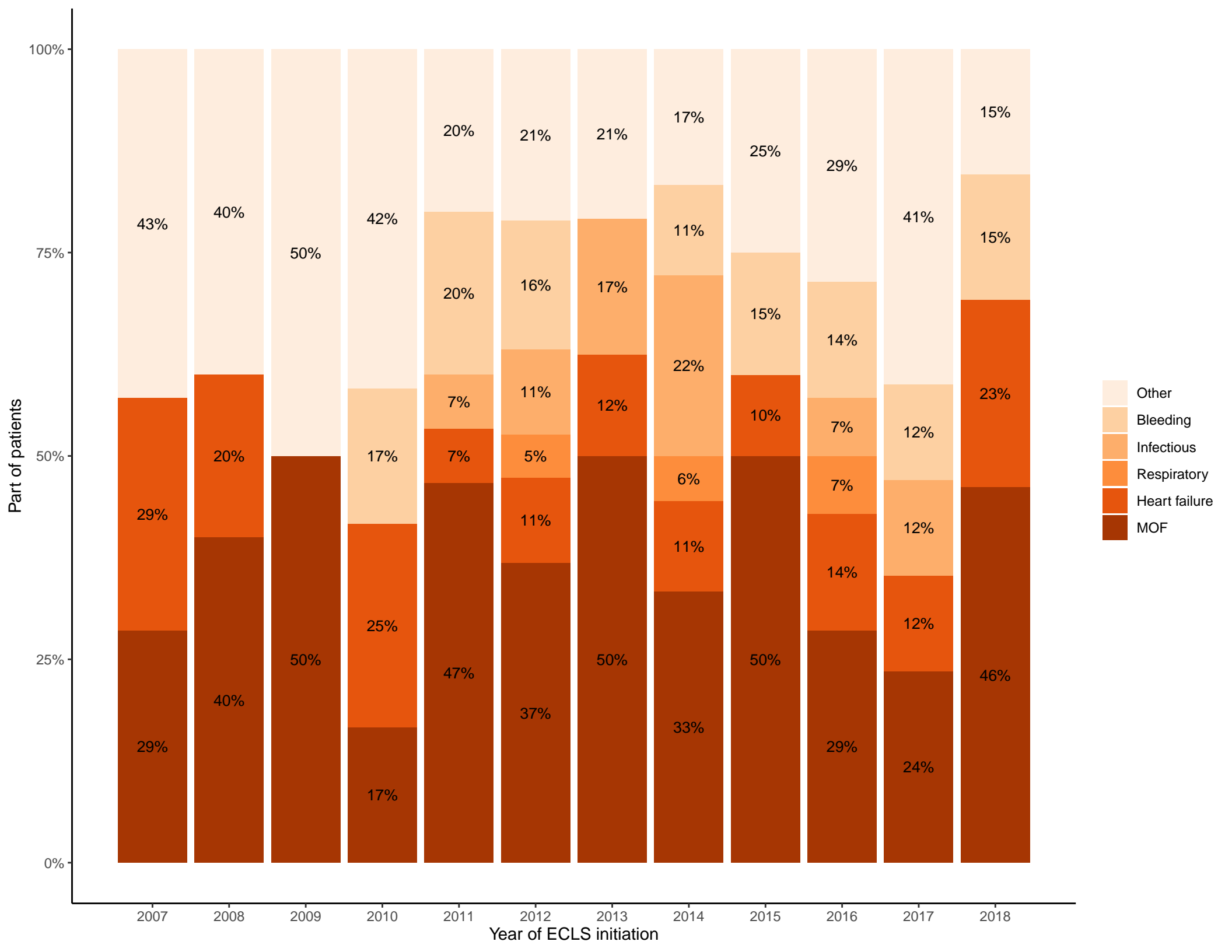

Supplement: Supplementary file 1 — Supplement, figure 5. Time to death (medians with interquartile ranges) and year of ECLS commencement [file 12471_2021_1552_MOESM1_ESM.pdf]

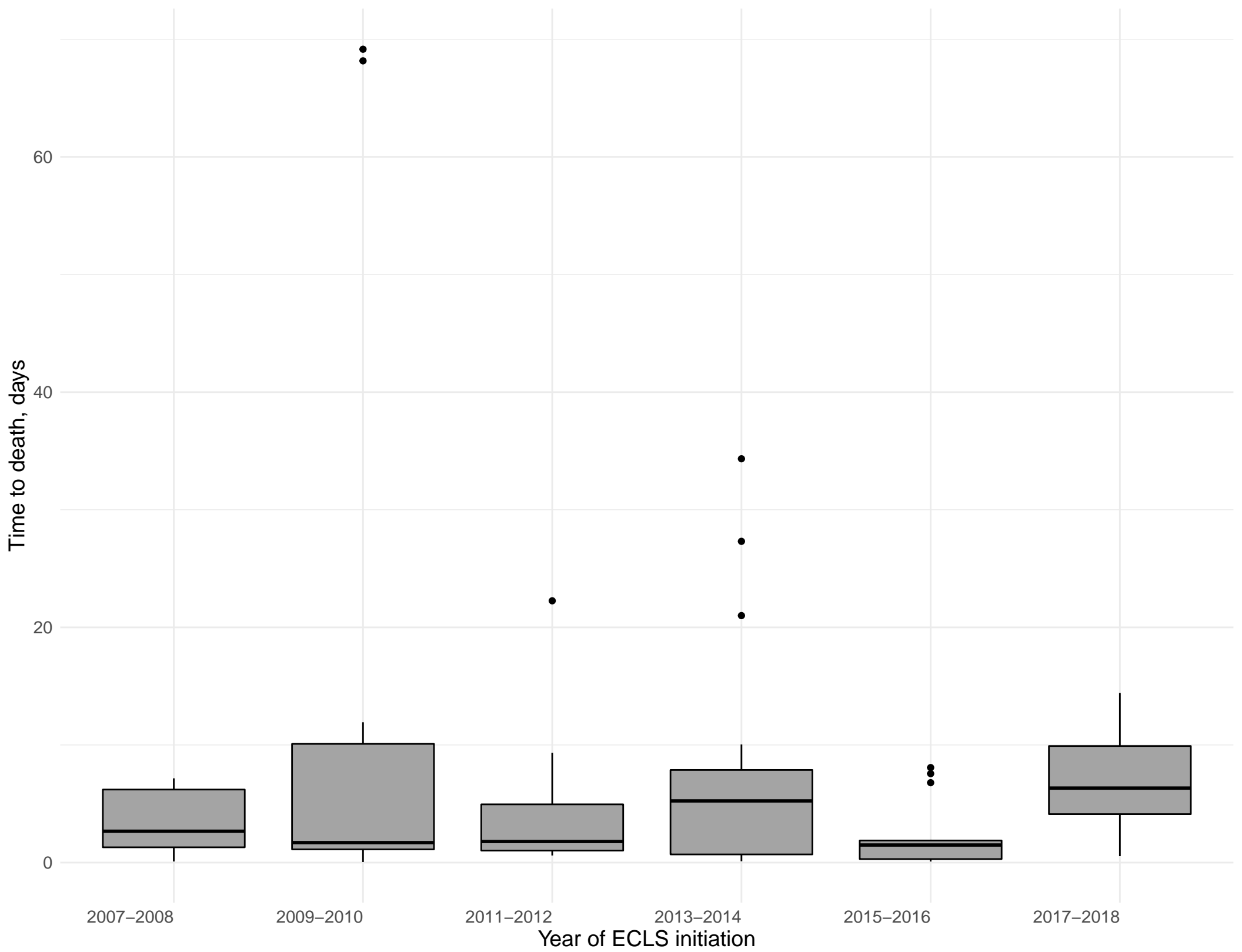

Supplement: Supplementary file 2 — Supplement, figure 6. Causes of death across year of ECLS initiation [file 12471_2021_1552_MOESM2_ESM.pdf]
